# Cup‐like blasts in acute myeloid leukaemia with *NPM1* and *IDH2* mutations

**DOI:** 10.1002/jha2.200

**Published:** 2021-05-06

**Authors:** Eiko Ohya, Yuka Sugimoto, Ryota Sasao, Misa Nishimura, Minoru Mizutani, Takao Sekine

**Affiliations:** ^1^ Department of Haematology and Oncology Matsusaka Chuo General Hospital Mie Japan; ^2^ Department of Community Haematology Mie University/Takeuchi Hospital Mie Japan

An 86‐year‐old woman presented with fever and pneumonia. Her laboratory investigations showed leucocytosis (15 × 10⁹/L) with 92% blasts, anaemia (haemoglobin 5.5 g/dl), thrombocytopenia (platelet 31 × 10⁹/L), and elevation of fibrinogen degradation products (FDPs > 120 μg/ml). The patient refused bone marrow examination. Hypogranular blasts without Auer rods containing nuclear invagination, the so‐called “cup‐like” or “fish‐mouth” appearance, accounted for approximately 17% of the blasts in peripheral blood. Blasts were strongly positive for myeloperoxidase and showed normal female karyotype (46,XX) [Figure 1]. The immunophenotypic features of the blast included expression of CD13(dim), CD33 and CD56 and absence of CD34 and HLA‐DR expression, which mimicked those of acute promyelocytic leukaemia (APL) [Figure 2]. Targeted next‐generation sequencing using the Oncomine Myeloid Research Assay (Thermo Fisher Scientific, MA, USA) revealed pathogenic variants with allele frequencies greater than 1% in *nucleophosmin 1* (*NPM1*; p.Trp288CysfsTer12), *isocitrate dehydrogenase 2* (*IDH2*; p.Arg140Gln) and *Fms‐like tyrosine kinase 3* (*FLT3*; p.Asn676Lys), of which the variant allele frequencies (VAF) were 47.94%, 51.3%, and 3.55%, respectively. The patient was eventually diagnosed with acute myeloid leukaemia (AML) with mutated *NPM1*.

**FIGURE 1:**
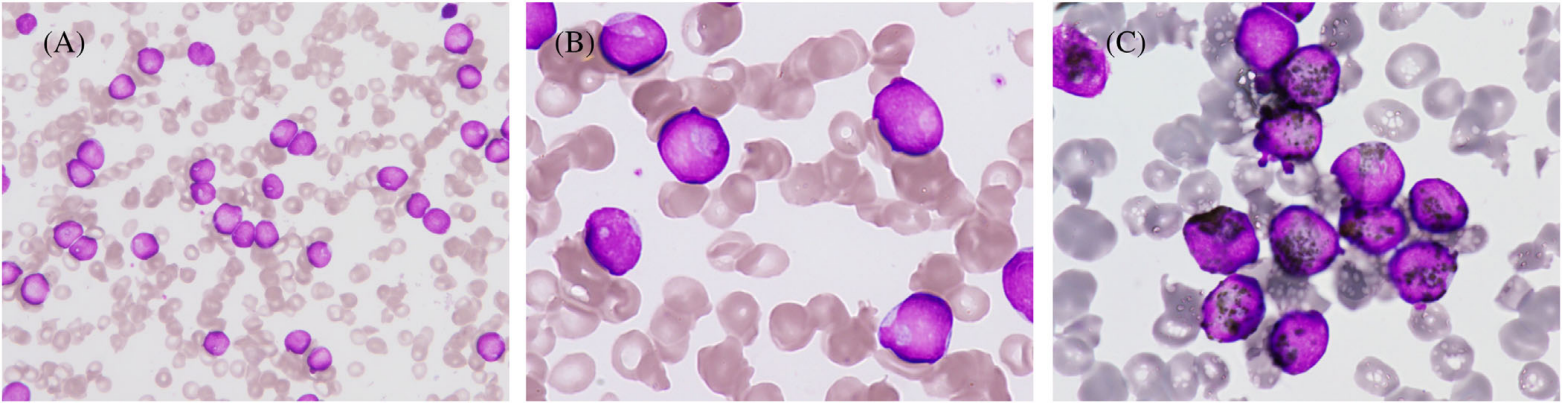
Morphological characteristics of the blasts in peripheral blood. Wright‐Giemsa stain (a and b), and myeloperoxidase stain (c). Original magnification: a, 400×; b and c, 1000× (oil immersion). Hypogranular blasts in peripheral blood had cup‐like nuclear indentation and strong myeloperoxidase positivity

**FIGURE 2:**
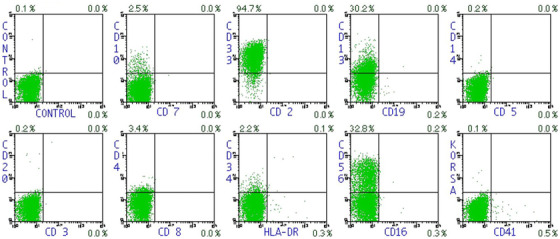
Flow cytometric analysis of peripheral blood cells. The blasts were positive for CD13 (dim), CD33, and CD56 and negative for CD34 and HLA‐DR

AML with cup‐like blasts (CLB‐AML) is defined as the presence of more than 10% of blasts with nuclear invaginations that span at least 25% of the nuclear diameter, which are commonly associated with AML M1/2 (myeloid) and M4/5 (myelomonocytic/monocytic) in the FAB classification. *NPM‐1* and *FLT3‐ITD* mutations have been detected in more than 60% of CLB‐AML cases with normal karyotype. *NPM1‐*mutatd AML showing APL‐like immunophenotype, negative for both CD34 and HLA‐DR, often harbours co‐mutations in *IDH1/2* or *TET2* [1]. Furthermore, high D‐dimer/FDP levels, CLB, and APL‐like immunophenotype are frequently observed in AML with mutated *NPM1* harbouring *FLT3* or *IDH1/2* co‐mutations [2]. Our case is consistent with these clinical, morphological, and molecular features. VAF of *FLT3* mutation is less than 5% and much lower compared to that of *NPM1* and *IDH2*, which means that *FLT3* mutation may not affect the cup‐like morphology and clinical presentation in our case. *NPM1*‐mutated AML carrying concurrent *FLT3‐ITD* mutation especially with high (≥0.5) allelic ratio is well known to be associated with poor prognosis. Molecular analysis is very important in CLB‐AML with APL‐like phenotype in terms of predicting prognosis and determining the optimal treatment strategy including allogeneic stem cell transplantation or molecular targeted therapies such as FLT3 or IDH1/2 inhibitors.

## CONFLICT OF INTEREST

Y. Sugimoto has received honoraria from Novartis, funding from Takara Bio, and research support from Astellas, Kyowa Kirin, and Ono, and is an accepted researcher from Shojunkai Takeuchi Hospital. All other authors do not have any conflicts of interest to declare.
